# Early Onset Preeclampsia and Intrauterine Growth Restriction: A Case Report

**DOI:** 10.7759/cureus.33919

**Published:** 2023-01-18

**Authors:** Fahimah A Almuhaytib, Nihad A AlKishi, Zainab M Alyousif

**Affiliations:** 1 Medicine, King Faisal University, Al-Ahsa, SAU; 2 Obstetrics and Gynecology, Maternity and Children Hospital, Al-Ahsa, SAU

**Keywords:** proteinuria, hellp, iugr, early onset, preeclampsia

## Abstract

Preeclampsia is a life-threatening illness during pregnancy. The main signs of preeclampsia are high blood pressure and proteinuria. Most cases of preeclampsia occur in the third trimester after 32 weeks and affect nulliparous women. Preeclampsia can lead to many serious complications, including the death of both the mother and fetus. In this case report, we reported a case of a 34-year-old Saudi woman, gravid 6 para 4 + 1 abortion, at 22 weeks by ultrasonography (US). Complained of a rare condition of early preeclampsia with hemolysis, elevated liver enzymes, low platelets (HELLP) syndrome, and intrauterine growth restriction (IUGR) due to placental insufficiency in the second trimester of pregnancy This pregnancy was terminated due to maternal risks. In conclusion, we can consider the importance of blood pressure screening in the early stages of pregnancy in the first or second trimester.

## Introduction

Preeclampsia is a fatal condition that occurs during pregnancy and can affect both the mother and fetus [1]. The primary signs and symptoms of preeclampsia include hypertension, lower limb edema, proteinuria, and thrombocytopenia [1]. Preeclampsia affects many body systems and leads to several complications [1]. There are many risk factors for preeclampsia like null parity, family history of preeclampsia or gestational hypertension, smoking, obesity, and diabetes mellitus [1]. Preeclampsia mostly occurs in the third trimester of pregnancy [2]. About 5% to 20% of pregnancies with preeclampsia are complicated with severe fetal complications for both the mother and fetus [3]. HELLP syndrome is a severe form of complicated preeclampsia [3]. IUGR is a severe complication for fetuses in mothers with severe preeclampsia and HELLP syndrome [4]. Many studies showed that there is a significate link between preeclampsia and the development of IUGR [4,5]. Pregnant ladies with high blood pressure or preeclampsia have a high risk of IUGR [4,5]. Here, we reported a rare condition of early preeclampsia and HELLP syndrome complicated with IUGR.

## Case presentation

Our patient is a 34-year-old Saudi woman. She is gravida 6 para 4 + 1 abortion. The patient was pregnant in week 25 by the last menstrual cycle (LMP). The pregnancy was spontaneous pregnancy. All previous pregnancies were normal spontaneous vaginal delivery at full term and uncomplicated. She has no other medical issues. No history of hypertension, renal disease, diabetes mellitus, trophoblastic diseases, or antiphospholipid syndrome. Surgically, she had an appendectomy and dilation and curettage (D&C) for one year for spontaneous incomplete abortion.

The patient complained of minimal vaginal bleeding and passing tissues before two weeks followed by severe epigastric pain radiating to the back associated with vomiting, lower limbs pitting edema, severe headache, and blurred vision for 1 week. On the physical examination, the patient looked well and vitally stable. However, she had three high readings of BP. First, 148/105 mmHg. Second, 159/98 mmHg. Third, 140/86 mmHg. In the abdominal examination, the abdomen was soft, lax, and had no tenderness. In the pelvic vaginal examination, there was minimal bleeding.

For imaging, we ordered a US and fetal growth chart. US showed a sinThe gle visible fetus, breech liquor adequate, and placenta up posterior. In the US measurements in the fetal growth chart, there was two to three weeks’ difference in the gestational age between LMP and fetal measurement. In Table 1, the fetal growth measurement for the fetus.

**Table 1:** Fetal growth measurement

2D measurement	value	Gestational age
Estimated fetal weight (EFW)	477g ± 70 gram (g)	22 weeks
Biparietal diameter (BPD)	5.63 centimeter (cm)	23 weeks 1day
OFD	7.51 cm	-
Head circumference (HC)	20.75 cm	22 weeks
Abdominal circumference (AC)	17.52	22 weeks
Femur length (FL)	3.59	21 weeks
posterior ventricle Vp	7.02	-

For the laboratory investigation, we ordered complete blood count CBC, urine dipstick, and liver function test. Urinalysis revealed a protein level of 3+. In CBC, there were thrombocytopenia and low Hemoglobin. In the liver function test, elevated liver enzymes in the liver function test. In Table 2, the CBC for the mother, and in Table 3 liver profile for the mother.

**Table 2:** CBC of the mother

CBC	Value	Normal range
Hemoglobin (HBG)	8.1 grams per deciliter (g/dL)	11.6-15 g/dL
Red Blood Cells count (RBC)	3.61 x10^12^/Liter (L)	3.8 - 5.2 x 10^12^/L
Hematocrit (HCT)	26.4%	40%-50%
Mean corpuscular volume (MCV)	73.1 femtoliters (fL)	80-100 fL
Mean corpuscular hemoglobin (MCH)	22.4 picograms per milliliter (pg/mL)	27-31 pg/mL
Mean corpuscular hemoglobin concentration (MCHC)	31.6 g/dL	30-35 g/dL
White Blood Cell count (WBC)	6.51 x10^3^/µL	3.5-9.1 x10^3^/µL
Platelet count	103 x10^3^/µL	157-371 x10^3^/µL

 

**Table 3:** Liver profile of the mother

Liver profile	Value	Normal range
Aspartate transaminase (AST)	89.2 units per liter (U/L)	15-37 U/L
Alanine transaminase (ALT)	73.5 U/L	14-59 U/L
Total protein	48.5 gram per liter (g/L)	66- 83 g/L
Albumin	23.6 g/L	34-50 g/L

The patient was diagnosed with preeclampsia with HELLP syndrome and complicated with IUGR due to placental insufficiency. We initiated the management by giving Labetalol 100 mg orally to control the BP. Then, we decided to terminate the pregnancy to prevented serious complications and safe the mother life. That is because she was complain of many severe signs and symptoms that did not improve by medications. The patient agreed to the management plan after discussing it with her. Misoprostol 400 mcg sublingual q4h for a maximum of five doses was used to terminate the pregnancy medically. After two doses of Misoprostol, she gave birth vaginally. A male baby weighted 425 gram was resuscitated by a pediatrician doctor. Postpartum, mother’s blood pressure returned to normal after delivery. The laboratory test repeated. The hemoglobin was 9.2 and the platelet count was 144. Prenatal care ordered a histopathology study of the placenta, but it was not completed for unknown reasons. The baby lived for 24 hours. Then, the infant then developed Pseudomonas aeruginosa infection and died from respiratory distress syndrome. Doctors planned for regular follow up for the mother.

## Discussion

According to the American College of Obstetrics and Gynecology (ACOG), preeclampsia occurs after 20 weeks of gestation [1]. However, some studies reported the majority of cases to occur in the third trimester after 32 weeks and affecting nulliparous women [2]. The occurrence of preeclampsia in the first or second trimester is usually associated with trophoblastic diseases or antiphospholipid syndrome [1,2]. The pathophysiology of preeclampsia represents that abnormal placentation or placental insufficiency is associated with worse clinical outcomes, higher diastolic BP, worse renal function, and perinatal fetal death [2]. Most reported cases of early preeclampsia affected nulliparous patients with risk factors [6]. However, our patient was multiparous and did not have any risk factors. In most cases, the mother shows improvement within weeks [6]. Our patient, on the other hand, improved within hours of delivery. That meant our patient's pregnancy was extremely dangerous. The management of preeclampsia depended on Neonatal factors [7,8]. In patients with severe signs and symptoms of high BP, severe persistent epigastric pain unresponsive to medication, severe headache, thrombocytopenia, and high serum creatinine, we consider termination of pregnancy to reduce the mother's risk of developing life-threatening complications [7-9]. Also, neonatal factors are important to make a decision of termination of the pregnancy [9].

## Conclusions

From this case report, we can consider the importance of blood pressure screening in the early stages of pregnancy in the first or second trimester. Pregnant women with high blood pressure should be under more care. To prevent fetal complications for both mother and fetus. In this case study, we mention the important link between preeclampsia, HELLP syndrome, and IUGR. So, for each case of early preeclampsia, we should consider IUGER and instigate it. In this case study, we have a limitation. Our limitation in this case study is this study needs more investigation in postpartum for the mother, fetus, and placenta in order to identify the underlying cause of the early start of preeclampsia and develop future prevention strategies.
